# Answered and Unanswered Questions in Early-Stage Viral Vector Transduction Biology and Innate Primary Cell Toxicity for *Ex-Vivo* Gene Editing

**DOI:** 10.3389/fimmu.2021.660302

**Published:** 2021-05-28

**Authors:** Amanda Mary Dudek, Matthew Hebden Porteus

**Affiliations:** ^1^ Department of Pediatrics, Stanford University, Stanford, CA, United States; ^2^ Department of Pediatrics, School of Medicine, Stanford University, Palo Alto, CA, United States

**Keywords:** AAV (adeno-associated virus), genome-edited cells, hematopoietic stem cell, toxicity, hematopoietic stem cell (HSC) transplantation, viral vector, DNA damage response (DDR), parvovirus

## Abstract

Adeno-associated virus is a highly efficient DNA delivery vehicle for genome editing strategies that employ CRISPR/Cas9 and a DNA donor for homology-directed repair. Many groups have used this strategy in development of therapies for blood and immune disorders such as sickle-cell anemia and severe-combined immunodeficiency. However, recent events have called into question the immunogenicity of AAV as a gene therapy vector and the safety profile dictated by the immune response to this vector. The target cells dictating this response and the molecular mechanisms dictating cellular response to AAV are poorly understood. Here, we will investigate the current known AAV capsid and genome interactions with cellular proteins during early stage vector transduction and how these interactions may influence innate cellular responses. We will discuss the current understanding of innate immune activation and DNA damage response to AAV, and the limitations of what is currently known. In particular, we will focus on pathway differences in cell line verses primary cells, with a focus on hematopoietic stem and progenitor cells (HSPCs) in the context of *ex-vivo* gene editing, and what we can learn from HSPC infection by other parvoviruses. Finally, we will discuss how innate immune and DNA damage response pathway activation in these highly sensitive stem cell populations may impact long-term engraftment and clinical outcomes as these gene-editing strategies move towards the clinic, with the aim to propose pathways relevant for improved hematopoietic stem cell survival and long-term engraftment after AAV-mediated genome editing.

## Introduction

### The Efficacy of AAV in Genome Editing

Recombinant Adeno-associated virus (AAV) has been highly successful in gene-replacement therapy for monogenetic disorders. It has shown effectiveness in slowly or non-dividing cells such as the liver, retina or central nervous system because the extra-chromosomal non-replicative AAV genome is not diluted by cell division. However, for genetic diseases which manifest in cells that are highly proliferative such as the hematopoietic system, gene replacement therapy using AAV is not likely to be successful because of the dilutional effect. For highly replicative cells, such as hematopoietic stem and progenitor cells (HSPCs), long-term modification needs to occur by integrating into the genome. Permanent genetic modification of HSPCs then becomes a way to alter the function of the entire hematopoietic system because all cells of the blood and immune system are derived from HSPCs. While retroviral gene replacement has been curative in some gene-replacement strategies such as for treatment of X-linked SCID, malignant transformation due to the semi-random nature of retroviral integration (1–3) remains a concern. An alternative to semi-random integration by retroviral vectors is to use genome editing to integrate the transgene at a specific genomic location. The development of the highly-specific Cas9 endonuclease has catalyzed the development of highly efficient targeted integration strategies in HSPCs using genome editing and homology directed repair (HDR). Using Cas9 endonuclease to induce a double-strand break (DSB) by sequence-specific recognition through a guide RNA (4), we can force the cells to activate DSB repair, including by homologous recombination (an essential cellular repair pathway). In cases of homozygous genetic disorders, where both alleles of a gene contain a disease-causing mutation, by providing a DNA template to the cell which contains the corrective sequence flanked by regions of sequence homology in the presence of Cas9/guide RNA ribonucleoprotein complex (RNP) the cell will use the exogenous DNA donor as a template for homologous recombination, in a method termed HDR (5). The cell only uses the provided donor template (“donor”) if sufficient quantities are provided, on the order of hundreds-thousands. While early studies showed successful HDR using plasmid or DNA fragments as a DNA donor template (6), the highly sensitive nature of many cell types, especially stem cells, to naked DNA in the cytoplasm causes significant Type I interferon toxicity and precludes using naked DNA plasmids from being an efficient option as a DNA donor for development of clinical therapies. AAV vectors however, as relatively simple replication-defective parvoviruses containing only capsid proteins VP1, VP2, VP3 and an ITR-flanked transgene have proved to be ideal DNA delivery vehicles because they do not significantly activate the anti-viral response like naked DNA does. The mechanism is from the non-pathogenic AAV evolving to shield its encapsidated DNA from detection such that it efficiently is delivered to the nucleus and can serve as the template for HDR.

### What Cell Types Have Successfully Undergone HDR and What Are the Clinical Applications?

**Figure 1:**
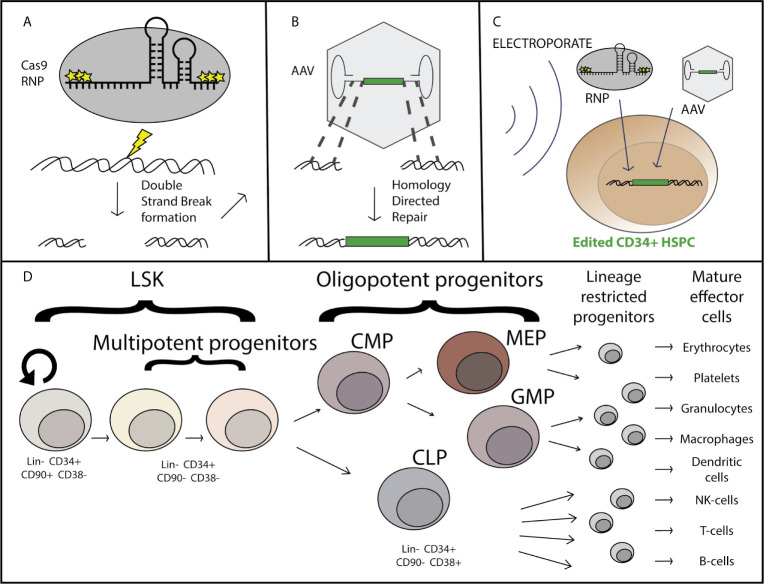
Overview of AAV-based hematopoietic stem and progenitor cell gene editing. Purified Cas9 protein complexed with chemically modified guide RNA as a ribo-nucleoprotein complex (RNP) induces a double strand break at a specific locus **(A)**, at which site flanking regions of homology within the recombinant AAV genome serve as templates for homology directed repair and incorporation of the corrective sequence [green] after DNA delivery by the AAV vector **(B)**. The genome editing components are delivered simultaneously *ex-vivo via* nucleofection the CD34+ hematopoietic stem and progenitor cells which have been stimulated to enter the cell cycle through treatment with a cytokine cocktail including SCF, TPO, Ftl3, and IL-6 **(C)**. The CD34+ subset of cells is comprised of both long term hematopoietic stem cells (LT-HSCs) which can self-renew and maintain full differentiation potential to reconstitute the entire hematopoietic system, as well as multipotent and oligopotent progenitors which have limited or no self-renewal capacity and are lineage restricted **(D)**.

Using this method of AAV-mediated genome editing by AAV delivery of a DNA donor for HDR (**Figure 1**), multiple groups have demonstrated high levels of gene targeting (the targeted insertion of a gene into a specific location in the genome, including single nucleotide gene changes) in many clinically relevant cell types, including HSPCs (5, 7–9), T-cells (10, 11), IPSCs (12, 13), mesenchymal stromal cells (14), and airway basal cells (15). Highly efficient (often >60%) allele targeting occurs in CD34+ HSPCs for blood disorders such as sickle cell anemia and beta-thalassemia (16, 17), immune disorders such as X-linked severe-combined immunodeficiency and chronic granulomatous disease (18–20), and lysosomal storage disorders such as mucopolysaccharidoses and Gaucher disease (21, 22). The transition from using plasmid DNA to AAV as a DNA donor was crucial for these genome editing therapies to reach high efficiency allele correction, due to the high toxicity associated with plasmid DNA in these highly sensitive primary cell types.

### What Are the Major Considerations for Continued Success of AAV-Mediated Gene Editing?

Successful development of AAV-mediated genome targeting is dependent on genome editing efficiency, genotoxicity, cell potency (including transplantability), and durability of the therapy. There have been many optimizations including cell culture conditions, nucleofection protocol, homology arm length in the DNA donor, guideRNA specificity, length of cell culture time, and the timing of the transduction of the AAV vector itself (23, 24). These optimizations have allowed highly efficient gene correction, demonstrated by as high as 90% allele targeting of the TRAC locus in human primary T cells (11). However, genotoxicity, transplantability, and durability of the gene-corrected cells after transplant are areas for continued improvement of these therapies to increase the probability of long-term safety and efficacy.

**Figure 2:**
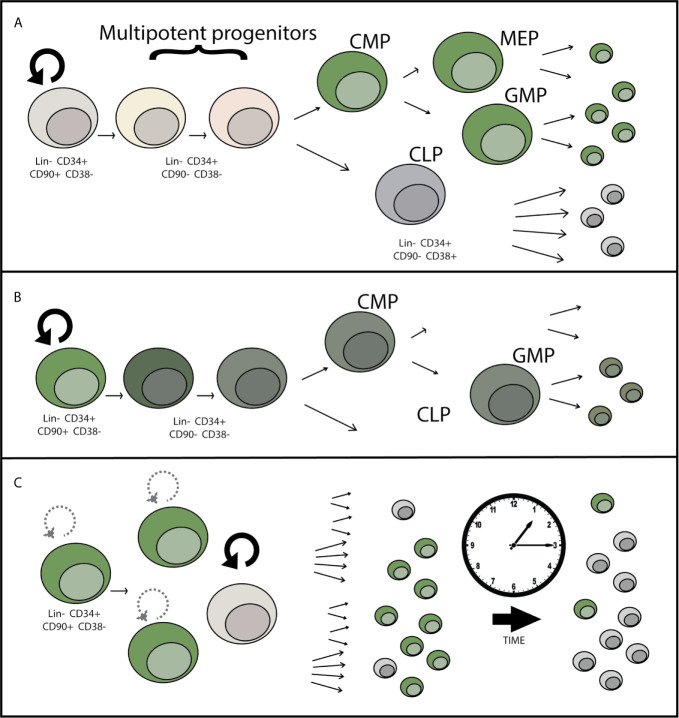
Potential mechanisms for loss of gene-edited HSPCs over time after engraftment. **(A)** LT-HSCs may be less permissive to HSC transduction than committed progenitors due to differential expression of AAV attachment, entry, or restriction factors. This potential progenitor-biased transduction would lead to dilution of the proportion of edited hematopoietic cells over time over time, as shorter-lived progenitor cells reach the end of their lifespan. **(B)** Toxicity within the LT-HSC or progenitor cell population in response to AAV causes a loss of differentiation potential or cell death leading to incomplete reconstitution of gene edited cells over time. **(C)** Decrease in the self-renewal capacity of LT-HSCs over time causes a loss of gene-edited LT-HSCs over time, eventually causing loss or near complete loss of the HSC population contributing to hematopoiesis that contains the genetic correction.

While AAV as a virus has evolved to avoid detection, data suggests that there can still be a residual detrimental effect in response to the AAV vector itself. Evidence that the AAV vector has toxicity is demonstrated by both measuring the response to the vector and by data showing that the toxicity increases as the amount of AAV used (multiplicity of infection or MOI) increases (24). While often genotoxicity is used to refer to aberrant DNA damage or mutations caused by the Cas9 endonuclease, the AAV genome burden within these cells can also be considered a type of genotoxicity. Single-stranded DNA (as in the AAV genome) normally only occurs in the cell in the context of viral infection or DNA damage, as revealed during the DNA repair process. When using anywhere between 5,000 and as much as 100,000 AAV vector genomes per cell for gene-targeting, these genomes can persist long past when targeted integration takes place. Although cells that have undergone AAV-mediated HDR can maintain their transplantability as shown by serial HSPC engraftment into NSG mice (16), we and others have shown that there is a significant drop in the efficiency of engraftment of gene-targeted cells compared to mock treated or RNP-only treated cells. This deficiency can be overcome through transplantation of 4-5 times as many cells (23), yet this is not always feasible when considering both the number of cells available to undergo editing, as well as from a manufacturing perspective. Therefore increasing/restoring the potency of engraftment after gene targeting would allow improvement in the successful implementation of this class of gene and cell therapies. In addition to transplantability, the durability of engraftment is an important consideration. Though bone marrow and peripheral blood HSPC transplants have shown long-term (life-time) durability and graft maintenance in human patients, loss of the overall proportion of gene-corrected cells over the course of the transplant in NSG mice suggests that there may be some type of defect related to AAV-mediated genome editing that either prevents efficient correction of the “true” hematopoietic stem cells (LT-HSCs), causes a loss of fitness of gene targeted progenitors, a defect in self-renewal of LT-HSCs, or a combination of these effects (**Figure 2**). As these therapies progress from pre-clinical animal models to clinical trials, monitoring the durability of gene targeted cells over time is essential in patients even if the initial gene correction and transplantation are successful. Because AAV-mediated genome correction and transplantation are the most advanced in HSPCs as compared to other stem cell types such as airway and induced pluripotent stem cells, this review will primarily focus on what we have learned from HSPC gene targeting and transplantation, potential areas of improvement, and the unanswered questions related to how the AAV vector interacts with the HSPCs and influences the long term success of gene-targeting therapies.

### What Are the Major Considerations for Successful Gene Editing Therapies in HSPCs?

When considering gene editing in HSPCs it is important to consider the functional and phenotypic differences in the HSPC populations. By definition, true long-term hematopoietic stem cells (LT-HSCs) have self-renewal capacity and have multi-lineage potential to reconstitute every cell-type of the hematopoietic system, including immune system cells (25). The LT-HSCs are primarily in a quiescent state *in vivo*, with the tremendous hematopoietic output (approximately 200 billion each of red blood cells, platelets, and neutrophils are made each day) deriving from progenitor cells. Progenitor cells have a limited self-renewal capacity, proliferate much more frequently, and become committed to a particular cell-type lineage within the hematopoietic system, the two predominant lineages being the myeloid and the lymphoid lineage. Immunophenotypically, LT-HSCs are currently defined by their cell surface markers (**Figure 1D**) as being Lin-, CD34+, CD90+, CD45RA+, and CD38-, while progenitors are defined as CD90-, CD34+, and CD38+ (26). Although immunophenotypic characterization is routinely done to define the HSPC subpopulations [for review see (27)], the current experimental gold-standard for determining LT-HSC function is through serial (2+) transplantation and multilineage hematopoietic reconstitution in immunodeficient mice with durability of engraftment shown for greater than 16-20 weeks and the true gold standard is engraftment and reconstitution in humans. For successful long term gene correction of cells in the hematopoietic system, correction of both LT-HSCs and progenitors is necessary as the progenitors are responsible for short-term blood and immune reconstitution early after transplantation, and correction of LT-HSCs is required for long-term correction as the shorter-lived progenitor cells are lost. While gene edited LT-HSCs have been maintained through sequential isolation and transplantation of human HSPCs and gene targeting of LT-HSCs has been confirmed through clonal tracking (28, 29), there is almost always a decrease in the proportion of corrected *vs* uncorrected alleles over course of the 16-20 week transplantation studies. This suggests that either true LT-HSCs are edited at a lower efficiency than progenitors, that edited cells are lost over time, or a combination of these possibilities.

This review will focus on what is currently known about the entry pathway and innate immune and DNA damage response activation in response to AAV, and what is known about how these effects may alter the viability of HSPCs in AAV-based ex-vivo gene editing therapies. We will focus on specific cellular mechanisms that have been implicated, and the perturbations thereof that have been shown to improve gene editing and/or cell survival outcomes with the hope of opening doors to decreasing cellular toxicity and improving clinical success in the stem cell gene editing field moving forward. This review complements the excellent review written by Kajaste-Rudnitski describing other aspects of the cellular response of HSPCs to genetic engineering (30).

## Body

### Does Entry Mechanism Influence Toxicity in Gene Editing of HSPCs?

#### What Is the Canonical AAV Entry Pathway?

**Figure 3:**
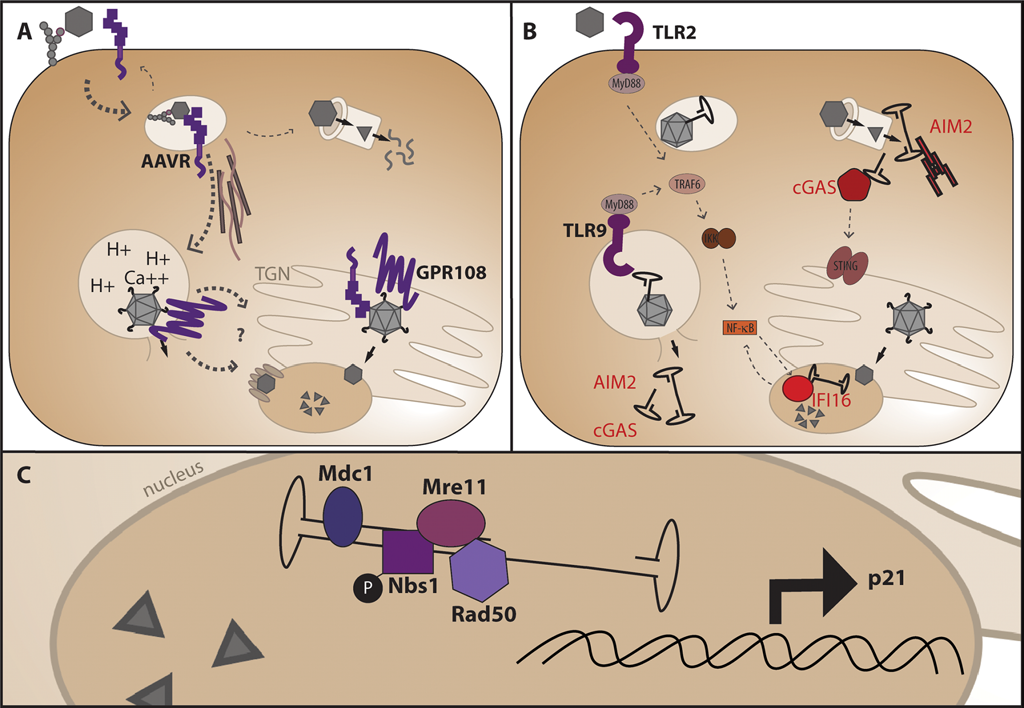
AAV entry mechanism and points at which genome release may trigger DNA-specific pattern recognition receptors. **(A)** After glycan attachment, the AAV capsid is endocytosed and binds the conserved cellular receptor AAVR, is trafficked along microtubules for productive infection through the endosomal system and TGN (below), or non-productive infection and degradation through the proteasome. Endosomal acidification and potentially other ions such as calcium facilitate a conformational change in capsid which releases the VP1 portion of capsid for interaction with the conserved entry factor GPR108 prior to nuclear import. **(B)** Where the genome is first sensed in the cell may dictate the cellular response and alter viability of HSPCs. Non-productive infection and capsid degradation through the proteasome may leave cytoplasmic AAV genomes available for recognition by AIM2 leading to apoptosis, or cGAS leading to NF-κB signaling *via* the adaptor protein STING. Genome extrusion in endosomes may activate TLR2 and/or TLR9 causing NF-κB activation *via* MyD88 signaling, and aberrant endosomal escape may also allow sensing by AIM2 or cGAS. Nuclear AAV genomes may be sensed by the nuclear DNA sensor IFI16 causing subsequent NF-κB signaling. **(C)** Nuclear DNA damage response proteins known to interact with the AAV genome during second-strand synthesis or expressed in response to AAV transduction.

AAV first comes into contact with the cell through serotype-specific glycans that facilitate attachment of the external surface of the VP3 portion of capsid to the cell (31–33) (**Figure 3A**). After attachment, the vector undergoes endocytosis and trafficking within the endosome towards the perinuclear region *via* microtubules (34), although the exact endocytosis mechanism (eg: clathrin dependent, clathrin independent, micropinocytosis) is still debated and may be cell-type and vector dose dependent (35–38). In addition to glycan attachment factors, the external surface of most AAV capsids aside from the unique AAV4 and AAVrh32.3 serotypes (39) engage with the AAV receptor, AAVR, one of the only required AAV entry factors conserved across different capsid serotypes and shown to be required *in vivo* (40). While AAVR can be observed at the surface of cell lines in culture, steady state subcellular localization and endocytosis time-course experiments suggest that a major function of AAVR as a receptor is to traffic the endocytosed AAV particle to the appropriate cellular compartment. After attachment and receptor binding, the capsid is known to undergo a conformational change in acidified endosomes in which the internalized VP1unique portion of capsid is extruded through the intact capsid exposing the phospholipase domain (41, 42) within this region thought to facilitate endosomal escape through cleavage of the lipid membrane. Recent identification of GPR108 as the second cellular entry factor highly conserved across capsid serotypes (43, 44) and required *in vivo* demonstrates an additional function for the VP1/2 unique region of the capsid in the entry pathway, as chimeric capsids and structural studies have demonstrated that while AAVR usage is dependent on the VP3 portion of capsid (45–49), GPR108 usage is dependent on the VP1/2 portion of capsid (44). Additional cellular factors may influence transduction efficiency either directly or indirectly such as the golgi-resident calcium transporter SPCA1, knockout of which demonstrates aberrant trafficking patterns within the cell (50). The recent identification and characterization of these golgi-resident factors over the past few years in addition to studies demonstrating that microinjection of AAV vectors into either the cytoplasm or nucleus does not facilitate transduction (51), highlight the importance of trafficking AAV through the endolysosomal system for productive and efficient transduction. While it is thought that AAV enters through the nuclear pore complex (52, 53), no nuclear pore proteins have been shown to interact directly with AAV capsid proteins as would be expected from a NPC-specific nuclear import mechanism and as seen with other NPC-using viruses, so questions remain about the exact nuclear import mechanism. Perhaps the most important and elusive question in the AAV entry pathway is at what point the AAV capsid disassembles and allows genome release. While we know the capsid must undergo conformational changes described above for transduction, and we know the capsid contains pores at the five-fold axes of symmetry large enough for the genome to fit through, it is unclear whether or not full capsid disassembly is required for genome release and if not where in the cell does the release occur.

#### What Is Special About AAV6?

Because AAV6 uses the same canonical factors AAVR and GPR108 as well as attachment factors shared by other AAV serotypes it is curious that AAV6 appears to transduce HSPCs for Cas9 mediated gene targeting so much more efficiently than other AAVs. Although there are reports that AAV capsid variants isolated from human HSPCs, termed AAVHSCs, can facilitate genome editing at higher frequencies than AAV6 in the absence of a specific DNA break (54, 55), this review is focused solely on AAV-based genome editing in the context of a Cas9-induced DSB so these capsids will not be reviewed at length there. Several groups have reported that AAV6 transduces HSPCs and other stem cell types higher than most other serotypes (56, 57), including the highly similar capsid AAV1 which differs by only 6 of the 737 capsid amino acids, for a sequence similarity of 99.2%. Indeed, we have reported that Cas9/AAV targeted integration at the *HBB* locus in HSPCs is 40% higher from AAV6 than from AAV1, while both episomal expression and targeted integration are 30-fold and 300-fold greater, respectively, from AAV6 than AAVHSC (58). The increased activity of AAV6 compared to AAVHSC in facilitating gene targeting in HSPCs following a DSB was also shown by the Cannon group (59). These 6 differential amino acids exist spread throughout the capsid as single amino acid substitutions, and these do not appear to come together to form any sort of unique domain in the fully assembled capsid (60). With the recent identification of the conserved AAVR and GPR108, it is possible that there are other entry or restriction factors specific to AAV6 in HSPCs that have yet to be discovered. It is also possible that in the absence of specific proteinaceous factors the capsid is inherently more efficient at uncoating or genome release in HSPCs therefore facilitating higher genome editing. In a sypro-orange thermostability assay, AAV6.2, the AAV6 capsid in which the unique amino acid in VP1 was reverted to the amino acid present in AAV1 (61), AAV6.2 demonstrates a significantly lower melting temperature than AAV1 suggesting it may be less stable than AAV1 (62). Interestingly, these capsids have the same melting temperature at very low pH of 3, yet the largest melting temperature difference is observed at neutral pH, so it is unclear how this melting data translates to the biological stability of these AAV capsids within the acidified endosome. While these details of AAV6 transduction in HSPCs may seem inconsequential as long as the genome reaches the nucleus, the process of how these AAV genomes reach the nucleus is of great interest both for improving genome editing efficiency, and in order to understand how and when the cell “see’s” the AAV and alters the cellular function within this highly sensitive, highly specific cell type. During the next portion of this review we will discuss points at which the HSPCs may sense the AAV vector and potential impacts this may have on the HSPCs based on what is known about innate immune and DNA damage responses in HSPCs. Although there have been many studies and reviews aimed at addressing how transgene products can cause immune recognition in genetic diseases which cause a complete loss of protein and destruction of transduced cells through CD8+ T cell reactivity to the AAV capsid *in vivo* (63), this review is focused on aspects inherent to the AAV vector (capsid proteins and AAV genome ITRs) in an *ex-vivo* genome editing context.

#### How Might the AAV Genome Be Sensed as a PAMP in HSPCs?

Innate cellular responses are activated by pattern recognition receptors (PRRs) that sense cellular stress through pathogen-associated or damage-associated molecular patterns (PAMPS or DAMPS). For an extended review of PAMP and DAMP recognition by PRRs, we refer readers to (64, 65), and this review will only discuss the pathways relevant to cellular damage caused by intracellular sensing of AAV vector components, namely the AAV ITRs. Additionally, while vector quality and purity from production is of extreme importance to minimize cellular contaminants that could be recognized as pathogen or damage associated, we will only discuss aspects inherent to the vector itself and not the production process. AAV as a minimal vector comprised of three capsid proteins and only ~145 bases of inverted terminal repeat AAV genome on either side of the transgene for packaging leaves little to be recognized by innate cellular responses. However, it is known that TLR9 which exists in the endosome of antigen presenting cells and recognizes dsDNA can be activated by AAV, signaling through the MyD88 pathway and causing amplification of CD8 T cell responses (66, 67) (**Figure 3B**). TLR2 has also been implicated in innate activation in response to AAV vectors (68). While TLRs are classically thought of as PRRs specific to antigen presenting cells such as macrophages and dendritic cells, several reports have demonstrated TLR expression on human HSPCs (69–71). In HSPCs, TLR-specific agonists induce cell cycle entry and lineage-specific differentiation. While several studies have demonstrated the importance of TLR signaling *in vivo* for hematopoiesis in response to microbial infection, ex vivo studies highlight the innate TLR functionality present in HSPCs. Ex vivo TLR9 activation by CpG DNA can induce mouse common lymphoid progenitors (CLPs) to differentiate to dendritic cells in response to herpesvirus infection (72). Additionally, TLR2 activation of human CD34+ cells *in vitro* with Pam_3_CSK_4_ pushes cells towards a myeloid-biased lineage, generating CD11c+ monocytes and dendritic cells (71, 73, 74). CpG depleted AAV vectors are in development to decrease TLR9 activation by vector genome CpG di-nucleotides *in vivo* (75–77). Recent incorporation of TLR9-inhibitory sequences derived from human telomeric DNA sequence have additionally been used to counteract TLR9 sensing *in vivo* (78). If AAV vectors are able to activate TLR9 in HSPCs *ex-vivo*, CpG depletion or incorporation of TLR9 inhibitory sequences may be a useful strategy for both *in vivo* AAV-mediated gene delivery as well as *ex-vivo* AAV-mediated gene editing.

In addition to TLR activation, the palindromic ITR hairpin DNA sequences could be sensed by cellular DNA sensors such as cGAS, IFI16, or AIM2. cGAS was originally described as a cytoplasmic DNA sensor which binds to short stretches of dsDNA and DNA/RNA hybrids and catalyzes the synthesis of cyclic GMP-AMP, a second messenger which activates STING to induce interferon through IRF3 dimerization and NF-κB signaling. However, recent immunofluorescent experiments have demonstrated that endogenous levels of cGAS can be found in the nucleus of LT-HSCs and it is kept silent through a small circular RNA called cia-cGAS to prevent activation in quiescent cells, yet this circular RNA does not exist in committed progenitors (79). In contrast, IFI16 is thought to be nuclear localized (80) and has multiple reported functions including regulation of transcription in hematopoiesis, as well as recruitment and activation of STING in the presence of viral infection (80). Expression of IFI16 has been shown in CD34+ cells from human bone marrow, which upon induction of differentiation was only preserved in cells of the monocytoid lineage as determined by expression of CD14 (81). Additionally, IFI16 expression has been shown to be 4-fold higher in quiescent HSCs compared to more proliferative progenitors (82). While cGAS and IFI16 are most commonly associated with induction of an interferon response, they have additionally been shown to induce non-canonical cellular responses such as cell cycle arrest (83, 84) and apoptosis or pyroptosis (85) in certain cell types. In contrast to cGAS and IFI16, the canonical activation of AIM2 causes induction of the AIM2-inflammasome (86) resulting in maturation of IL-1β and IL-18 as well as a type of programmed cell death called pyroptosis (87). Interestingly, mice deficient in AIM2 are protected from bone marrow failure after total body irradiation (88), demonstrating that AIM2 plays a role in apoptosis induction in the hematopoietic system. While some of these DNA sensing proteins have similar canonical downstream effector proteins involved in IFN activation, protein complex differences in unique cell types can greatly influence the cellular outcome from activation. There have been reports that activation of certain IFI16 isoforms can inhibit AIM2 inflammasome formation (89), and AIM2 can inhibit cGAS by cleavage through caspase-1 (90). DNA virus induced caspase-3 has also been shown to cleave cGAS, MAVS, and IRF3 to prevent overproduction of inflammatory cytokines (91), demonstrating that there is cross-talk between DNA sensing pathways and suggesting that aspects of the cellular response can be post-transcriptionally regulated. It is currently unclear if the AAV ITRs activate these cytoplasmic DNA sensors and how differential activation may influence the outcome of gene-editing therapies in HSPCs, yet observed toxicity from the vector *in vitro* suggests there is an inherent response which may influence cell survival after editing and transplant.

#### Where Is the Genome First Sensed in the Cell?

When considering potential cellular responses to the AAV vector it is important to consider where the genome is first sensed in the cell, as the most likely component to be sensed as a danger signal. Herein lies the important distinction of entry pathway usage, as endocytosis is not synonymous with true viral vector entry (ie: breach of a membrane barrier to the internal cytoplasm or nucleus) and may differ in cell types which have differential cellular responses to the vector. It has been demonstrated by other viral vectors such as adenovirus that capsids which traffic through and accumulate in the perinuclear region in late endosomes (as demonstrated through co-localization with LAMP1 and M6P) induce inflammatory cytokines while Ad5 which traffics directly to the nucleus does not (92), presumably due to increased recognition by intracellular viral DNA sensors. A variety of AAV serotypes have shown similar peri-nuclear accumulation during the entry pathway and co-localization with Rab7 and Rab11 suggest trafficking through late and/or recycling endosomes (93), as well as through the Trans-Golgi network (TGN) (94). The AAV receptor AAVR has demonstrated functional necessity for trafficking to these compartments late in the endolysosomal system, as domain swap chimeras containing the AAV binding and transmembrane domains with the cytoplasmic tails of well-defined trafficking domains demonstrates that trafficking to the TGN maintains full AAVR function yet chimeras containing c-tails that traffic to early or late endosomes but not TGN have drastically reduced functionality (40). In addition to trafficking considerations, the GPR108 protein has been implicated in regulation of NF-κB signaling, with knock-out mice demonstrating higher cytokine production (95), yet it is unclear whether AAV association with GPR108 triggers its functionality within the NF-κB pathway or if it solely takes advantage of the protein to gain access to the cell without triggering its activity. It is well-known that AAV can be degraded through the proteasome (96), and decreased efficiency of trafficking in some cell types may increase the amount of proteosomal degradation leading to increased availability of AAV genomes for intracellular recognition and a magnified cellular stress response. Additionally, when considering that vector copy numbers of 5,000 to 100,000 genomes per cell are used for transduction and genome targeting, with hundreds or thousands of vector genomes per cell isolated from cytoplasmic extracts of transduced cells in order to achieve a functional multiplicity of infection (MOI) of 1 (meaning on average 1 functional transduction event per cell for all cells to have undergone successful gene replacement or gene targeting) a major question remains about what happens during non-productive infection and how are these extra hundreds/thousands of genomes being sensed and responded to within the cell. While there is great focus on genotoxicity caused by DNA break induction from Cas9 during genome editing, a source of genotoxicity especially in sensitive stem cell populations is likely caused by the genotoxicity of lingering AAV genomes from non-productive transduction events after uncoating or capsid degradation.

#### How Might the AAV Genome Be Sensed as a DAMP in HSPCs?

While lingering non-productive vector genomes may be detrimental the cell, an additional source of cellular stress may be during productive transduction when the genome is sensed in the nucleus. As a DNA hairpin with a free DNA end, the ITRs can also be sensed by DNA damage response (DDR) proteins in the nucleus (97). It has been demonstrated through co-localization experiments that a variety of DDR proteins co-localize with nascent vector genomes upon nuclear entry as they undergo second-strand synthesis in discrete nuclear foci. These proteins include Nbs1, phosphorylated NBS1 (p-S343-Nbs1), Mre11, Rad50, and Mdc1 (**Figure 3C**). The Mre11-Rad50-Nbs1 complex is responsible for recognizing dsDNA nicks near the 5’end of a DSB to facilitate end resection required for DSB repair through homologous recombination. The co-localization of these proteins within the nucleus is interesting, as it begs the question whether this recognition is harmful as the sensing of a DNA break, or helpful by recruitment of HR machinery during AAV-mediated HDR. Because the choice of HDR compared to the error prone non-homologous end joining (NHEJ) pathway is dictated by successful end resection (98) it is possible that presence of these proteins may be helpful for skewing the repair pathway post-break towards the HDR pathway in the presence of AAV. Exposure of cells to DNA-damaging agents which upregulate DDR proteins has been shown to increase recombinant AAV vector transduction in the absence of Adenovirus co-infection (99–101), yet the mechanism is not fully understood and it is unclear whether a similar effect would be observed in gene editing applications where transgene expression is driven after targeted integration rather than from the episomal genome. Although gene expression can be observed from episomal AAV through either inherent promoter activity in the ITRs or concatemeric AAV genomes (102–105), the highly proliferative nature of HSPCs causes a quick loss of this expression through a dilutional effect of unintegrated AAV genomes (5) and transcriptional activity may be regulated differently from episomal AAV compared to integrated DNA sequence after HDR. It has also been demonstrated that AAV transduction in HSPCs and other cell types upregulates p21 expression (24, 106, 107), and addition of the ATM-kinase inhibitor KU55933 has been shown to decrease the number of apoptotic HSPCs after AAV transduction (107). Addition of GSE56, a p53 dominant negative mRNA rescues engraftment defects after AAV/Cas9 gene editing when co-electroporated with the genome editing components (24), yet it is unclear if this inhibitor would counteract the loss of engraftment potential seen in HSPCs from AAV alone. Because the p53/p21 pathway influences many aspects of cell function including cell-cycle arrest, apoptosis, senescence, and suppression of HR, this activation could be affecting HSPC function in many different ways. Interestingly, it has been shown that p53-null cells undergo apoptosis after transduction with AAV (108), so it is clear there are aspects of the cellular response to AAV that are caused by p53 activation. While it is clear that there are many interactions of the AAV genome with cellular replication and DNA damage response proteins, it is unclear how AAV genome recognition dictates the cellular response after recognition.

While presented here as separate cellular responses, the innate immune and DNA-damage response pathways are inextricably linked. A major function of cellular DNA sensors is to prevent cellular replication in response to not only viral infection but to DNA damage as well and the cellular outcomes are determined by the mechanism and magnitude of the cellular response. The cGAS/STING pathway gets activated as a response to genotoxic stress due to DNA damage, and the magnitude determines whether cells will repair, go into senescence, or undergo cell death (109–111). In addition, IFI16 has been reported to negatively regulate p53 and p21 to influence p53-mediated cell cycle arrest (83, 112). In the following section we will discuss what can be learned from replication of other parvoviruses within the hematopoietic compartment, as well as what has been learned so far from initial investigations into pathways activated by AAV transduction in HSPCs

#### What Cell Intrinsic Effects Are Known to Occur in Response to Parvoviral Infection in HSPCs?

**Figure 4:**
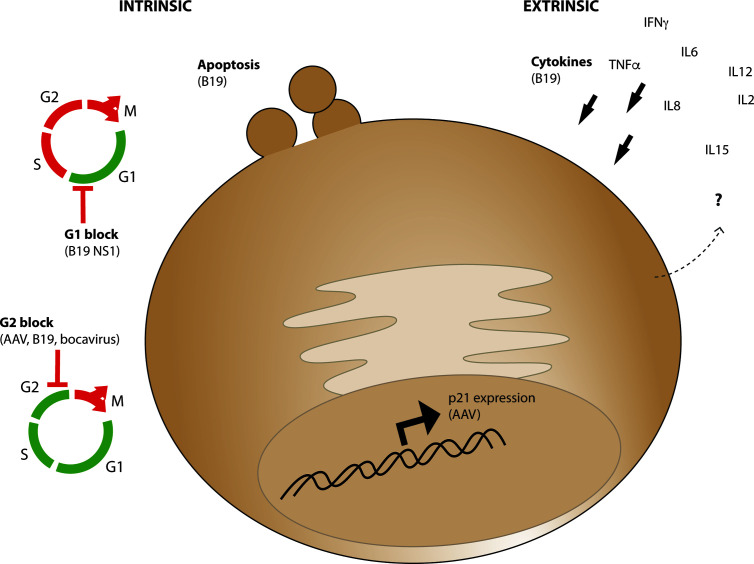
Intrinsic and Extrinsic parvoviral effects which may influence CD34+ cell survival and functionality. Intrinsic cellular effects (left) observed from AAV or other parvoviruses include apoptosis, G1 cell cycle arrest through B19 parvovirus NS1 expression, or G2 cell cycle block from AAV, B19, and bocavirus genomes. Extrinsic effects (right) observed from parvovirus B19 infection include expression of cytokines which promote CD34+ cell differentiation including IL6, which are expressed from various cell types *in vivo* during acute and chronic infection.

The HSPC population must undergo strict regulation to mobilize blood and immune cells in response to a variety of infectious agents, yet few viruses affect them directly. Parvoviruses, of which AAV is part of the dependoparvovirus subfamily, are one of the few types of viruses which can actually infect cells of the HSPC lineage. Human Parvovirus B19 has a selective preference for cells of the erythroid lineage within the bone marrow and can cause both transient and persistent erythroid aplasia, anemia, and bone marrow failure. B19 can cause apoptosis, G1 cell-cycle arrest, and G2 cell cycle arrest in target cells (**Figure 4**) and apoptosis and G1 arrest are a result of toxic genomic NS1 expression after entry (113). AAV Rep protein exerts analogous functions in virus replication to NS1, yet is provided in trans during vector production and is not present in the AAV vector after purification or encoded in the vector genome. Rare packaging of rep-containing DNA sequences have been observed in vector preparations (114, 115), yet packaging of undesirable genome elements such as rep-containing plasmid sequence or human sequence are rare (116) and prevalence of aberrant genome packaging likely is influenced by transgene sequence (117). However, it has been demonstrated that G2 cell-cycle arrest can be induced by both live and UV-inactivated B19, AAV, and bocavirus (118) suggesting that a major cause of cellular toxicity is simply the presence of parvovirus genome even in the absence of replication. This toxicity was shown to be caused by the bocavirus terminal repeats (119), and the AAV ITRs through nuclear injection of the AAV ITR sequence in human embryonic stem cells which causes apoptosis (120). Importantly for AAV vector considerations, this toxicity was observed in response to purified full but not empty capsids, demonstrating that toxicity in stem cells is due to the presence of encapsidated vector genomes, not cellular contaminants from the production process or from the capsid itself. While the replication competent B19 causes extreme hematopoietic toxicity and WT AAV infection has no known human pathology despite anti-capsid antibodies demonstrating common infection in humans (121–123), by investigating commonalities within this virus family we may identify points of intervention at which the health and survival of HSPCs after AAV-mediated gene editing can be improved.

Preliminary studies have aimed to identify transcriptional changes induced in HSPCs after editing by microarray gene expression profiles after treatment of cells with the individual gene editing components including AAV treatment (124). 24 hours after electroporation and AAV transduction HSPCs upregulate apoptosis factors DHRS2, GZMB, GDF15, and CCL2, as well as stress response genes RAP1GAP and BICC1. Transduction alone specifically upregulates DDR proteins RPA4 and PHLDA3, stress response proteins RAP1GAP and BICC1, and the immune molecule CD86. Of note, RPA has previously been implicated in AAV genome replication (125) and PHLDA3 is a p53-regulated Akt suppressor, deletion of which decreases p53-dependent apoptosis (126). These data are suggestive that AAV transduction of HSPCs induces an apoptosis program in some cells. Additionally, upregulation of CD86 which is normally expressed on antigen presenting cells such as dendritic cells and monocytes but not HSPCs suggests that AAV transduction pushes cells toward a lineage committed state and decreases regenerative potential. While it remains to be determined the mechanism by which AAV is altering hematopoietic stem cell fate, it is clear that there is a transcriptional response occurring that may alter HSPC function.

#### What Cell Extrinsic Effects Are Known to Occur in Response to Parvoviral Infection in HSPCs?

The tight regulation dictating hematopoiesis is determined by cytokines in the blood and bone marrow niche which determine the proliferative, regenerative, and differentiation fate of the stem and progenitor cells in response to a variety of stimuli including infection. The bone marrow niche plays a huge role in this regulation, but cytokines in ex vivo culture play a major role in the survival and function of HSPCs as well. In culture, a combination of cytokines usually including stem cell factor (SCF), thrombopoietin (TPO), Fms-related tyrosine kinase 3 ligand (Flt-3 ligand), and interleukin-6 (IL-6) are used to both maintain HSPC survival as well as push quiescent HSCs into the cell cycle in order to express the genes required for successful homologous recombination. Studies of parvovirus B19 infection *in vivo* have demonstrated increased levels of cytokines known to influence HSPC survival and differentiation including interferon-γ, tumor necrosis factor-α, IL-6, and IL-8 (127). Early infection at the initial peak viral load during acute infection has also demonstrated elevated IL-2, IL12, IL-15 levels in the absence of IFN-γ, while persistent infection was associated with elevated IFN-γ (128). Because these data were collected from patients it is presumed these cytokines were produced from B19 responsive CD8+ T cells (129, 130). However, it has been shown that LSK cells have the ability to produce a variety of cytokines including IFN-γ, IL-6, IL-2, IL-12 at high levels in response to danger signals (131). Single-cell analysis of multipotent progenitors (MPPs) demonstrated that 69% of cells produced at least one and up to 12 different cytokines in response to TLR stimulation by LPS and Pam_3_CSK_4_ (131), a TLR-2 ligand previously shown to activate TLR-2 in human CD34+ cells (71, 74). It is unclear if HSPCs directly express cytokines in response to parvoviral infection with B19 or AAV transduction during gene editing, but if so then paracrine functions on neighboring cells in culture may influence the engraftability of cells after editing.

Inflammatory cytokines are well known to modulate the hematopoietic compartment, and play important roles in mobilization from the bone marrow (132–134). Tight regulation of HSC response to inflammatory cytokines through cellular factors such as interferon regulatory factor-2 (IRF2) and ADAR1 are essential for the suppression of HSC exhaustion due to interferon signaling (135, 136). Factors such as TGF-β differentially regulate distinct hematopoietic stem cell subtypes; even true LT-HSCs are thought to be a somewhat diverse cellular population with individual cells being biased towards the myeloid or lymphoid lineage, and this differentiation is thought to be regulated through TGF-β (137). Conflicting reports on the effect of IFN-γ demonstrate an obvious but poorly understood effect of IFN-γ on hematopoiesis (138). Some reports have suggested that treatment of HSPCs with IFN-γ in culture causes expression of pro-apoptotic genes and reduced progenitor survival in a colony forming assay, yet a conflicting report suggests that IFN-γ exposure increases cell viability and colony formation. In vivo, IFN-γ injection has been shown to increase progenitor proliferation and activate dormant hematopoietic cells. Based on these experiments, it is suggestive that low cytokine levels leading to low levels of immune activation may increase survival of HSPCs, while high levels of activation are detrimental and lead to exhaustion of the hematopoietic compartment.

## Discussion

### How Do These Cellular Responses to AAV Alter the Long Term Potential and Engraftment of HSPCs?

The DNA damage and innate immune response pathways have integral interactions within them that influence cellular responses (139). This leads us to a chicken and egg problem about what cellular response comes first in response to AAV transduction of HSPCs and what is the rotten egg that is detrimental to HSPC engraftment after AAV-mediated gene editing. A variety of advances have been made to improve gene-editing efficiency and specificity in HSPCs including transition from using Cas9 mRNA to purified Cas9 RNP complex, chemical modification of gRNAs to decrease immune sensing (140), optimization of homology arm and guide RNA specificity, engineering of high fidelity Cas9 nucleases (141), increasing the purity of AAV vector preps, and decreasing the amount of AAV used. In addition, a variety of small molecules have been used to try to boost HDR efficiency during genome editing (142). These advances have led to extremely specific and efficient AAV-mediated genome targeting in many primary cells, and now the major area for improvement is increasing cell potency and viability during editing and engraftment after AAV transduction.

### How Do DDR and Innate Immune Pathway Activation Alter Engraftment Efficiency of HSPCs?

A lot can be learned about HSPC function from examining genetic diseases which cause a defect in components of the DDR pathway. Patients with defects in DNA damage signaling and repair [such as in ataxia-telangiectasia (A-T) and Fanconi-anemia (FA), respectively] highlight the importance of these pathways in HSCs as these patients have a high incidence of bone marrow failure and hematological malignancies. Competitive reconstitution experiments in mice demonstrate that deletion of a variety of different DDR pathway proteins cause a defect in hematopoietic reconstitution. Cells transplanted from knock-out mice demonstrate a decrease in progenitor cell reconstitution relative to WT cells, while young mice have no defect in overall HSC number suggesting this defect is a proliferative or regenerative defect, not a HSC developmental defect. Additionally, macrophages deficient in FA proteins have an increased production of inflammatory cytokines in response to TLR ligands, while the progenitor cells are hypersensitive to these cytokines which include IFN-γ, TGF-β, and IL-1. Though the mechanisms are still debated it is clear that these pathways are intricately related and greatly influence HSPC function and survival *in vivo*. There appears to be a balancing act for the level of innate immune activation to promote HSC quiescence and self-renewal compared to activation and differentiation (143). The proposed mechanism of the self-renewal improving small-molecule UM171 is through low level activation of inflammation but high concentrations of UM171 are detrimental to cell survival (144, 145), supporting this balancing act model. Several reports have demonstrated that low-level NF-κB activation promotes quiescence and self-renewal of LT-HSCs, suggesting that some level of innate response is beneficial to the survival and function of these cells.

### What Methods Have Been Used to Improve Engraftment After AAV-Mediated Genome Editing, and Where Do We Go From Here?

There are currently no specific targets known to increase efficiency of engraftment specifically after AAV transduction. During *in vivo* engraftment experiments, the main method to increase engraftment is by transplanting more cells but as these studies move from small animal (NSG mouse) to human studies the feasibility of transplanting higher numbers of edited cells becomes limiting. In addition to transplanting more cells, inclusion of several cytokines and small molecules such as UM171 in culture throughout the course of editing are known to improve engraftment (23, 144, 146). Factors which support stem cell survival such as stem cell factor (SCF) and thrombopoietin (TPO) greatly enhance the engraftment potential of HSPCs when maintained in culture. However, the benefit of including these cytokines occurs in all transplant groups, not just when transplanting AAV-transduced cells. While the incorporation of GSE56 during AAV-mediated genome-editing has been shown to preserve engraftment efficiency and increase clonality of transplanted cells (24), it is of great importance to further understand specific pathways which can be safely manipulated in the absence of potential unwanted cellular responses. Over 50 years of hematopoietic stem cell research has taught us much about HSPC biology, yet we have little understanding of how AAV transduction alters the function of HSPCs and their health and survival during *ex-vivo* AAV-mediated genome editing. It is likely that the first-in-human clinical trials using AAV6 as a donor for gene correction, will teach us even more about the potency of gene-targeted HSCs and serve as the basis for further optimization. Nonetheless, as this novel class of genetic medicines moves towards the clinic, continued rigorous study of the basic biology in primary cells such as HSPCs to determine pathways activated and altered by AAV transduction will allow for the improved success of AAV-mediated gene editing in the future.

## Author Contributions

AMD and MHP conceived and assembled information for this review. AMD wrote and generated figures, and MHP contributed significant edits and intellectual content. All authors contributed to the article and approved the submitted version.

## Funding

Grant support for AMD is through NIH F32 grant number 1F32HL154667-01. MHP thanks the Doris Duke Charitable Trust (#22399) and the Sutardja Chuk Professorship for support of this work.

## Conflict of Interest

MHP holds equity in CRISPR Tx, Graphite Bio, and Allogene Tx; serves on the Board of Directors of Graphite Bio and SAB’s of Allogene Tx and Ziopharm Oncology Inc. and receives compensation for those activities.

The remaining author declares that the research was conducted in the absence of any commercial or financial relationships that could be construed as a potential conflict of interest.

## Abbreviation

AAV, Adeno-associated virus; AAVR, AAV receptor; CLP, common lymphoid progenitor; CMP, common myeloid progenitor; DAMP, damage-associated molecular pattern; DDR, DNA damage response; DSB, double-strand break; GMP, granulocyte monocyte progenitor; HDR, homology directed repair; HR, homologous recombination; HSPC, hematopoietic stem and progenitor cell; ITR, inverted terminal repeat; LT-HSC, long-term repopulating hematopoietic stem cell; MEP, myeloid erythroid progenitor; NHEJ, non-homologous end-joining; NPC, pnuclear pore complex; PAMP, pathogen-associated molecular pattern; PRR, pattern recognition receptor; RNP, ribonucleoprotein complex; TGN, Trans-golgi network.
